# Association Army and Navy Medical Officers of the Southern Confederacy—An Appeal to the Physicians of Texas

**Published:** 1902-01

**Authors:** 


### Association Army and Navy Medical Officers of the Southern Confederacy—An Appeal to the Physicians of Texas.

DALLAS, TEXAS, December 1, 1901.

The Association of Medical Officers of the Army and Navy of the Confederacy has honored Texas with its next meeting, April 22 to 25, 1902, and in order to give these old veterans an appropriate reception will require several thousand dollars.

The membership of this association is as follows, as per extract from the constitution:

"All members of the medical profession who served as surgeon, assistant surgeon, contract physician, or acting assistant surgeon, hospital steward, or chaplain, during the late war between the States, shall be eligible to membership as members, and the secretary shall be instructed to enroll their names as such when application in writing is furnished, together with a statement of the official position and rank held in the army or navy of the Confederacy by the applicant.

"All Confederate veterans who are regular doctors of medicine are eligible to membership as associate members, and all sons of

Confederate veterans who are regular doctors of medicine shall be eligible to membership as junior members."

As chairman of the Finance Committee, I make this appeal and ask you to contribute according to your ability. If there is not a member of the Finance Committee in your vicinity, you can remit direct to me or to National Bank of Commerce, Dallas, for this fund.

Yours truly,

A. M. Elmore, M. D.,

Chairman Committee.

---

El Paso, Texas, December 19, 1901.

*Dr. F. E. Daniel, Editor Texas Medical Journal, Austin, Texas.*

Dear Doctor: I enclose some resolutions passed by our medical society which, if you think best, you can publish in your journal. We recognize that the "Red Back's" arguments against our town are good, and hard to answer, and for the good of the State Association we are willing to give up for this time, with the hope that you will be with us some future time.

Yours truly,

J. A. Rawlings,

Secretary El Paso County Medical Society.

The "Red Back" favors meeting in Fort Worth, because, on the same days, April 22 to 25, the Confederate Veterans will hold their reunion in Dallas, and also the Army and Navy Medical Officers; and Dallas will be crowded to overflowing, and accommodations for the State Medical Association will be impossible. Or else, let the President change the date of meeting, and let us meet in Dallas—avoiding a clash with the C. V.'s.

